# Supplementary material for: Conditional chemoconnectomics (cCCTomics) as a strategy for efficient and conditional targeting of chemical transmission
Source: eLife. 2024 Apr 30;12:RP91927. doi: 10.7554/eLife.91927 (PMC11060718; doi:10.7554/eLife.91927)
Supplement: Figure 3—source data 3. [file elife-91927-fig3-data3.zip › Figure 3 source data3 part 2.zip!/Figure 3-Source data 3-part2/1060-9-CRISPResso_Report_q0_g4o36.zip!/CRISPResso_on_1060_12/9.Alleles_frequency_table_around_sgRNA_gtcctacgtctggtgggtca.pdf]

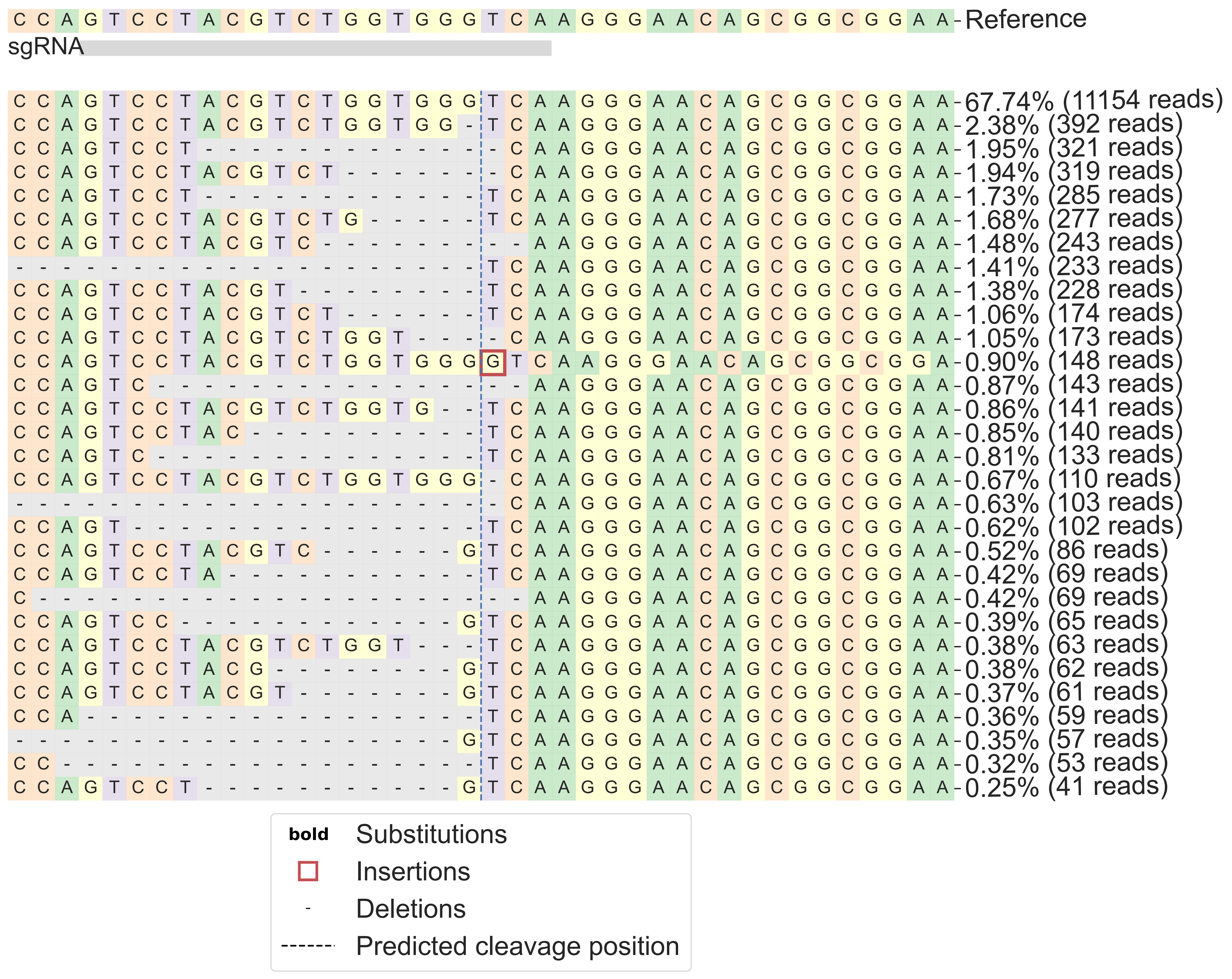

CCAGTCCTACGTCTGGTGGGTCAAGGGAACAGCGGCGGAA Reference
sgRNA
CCAGTCCTACGTCTGGTGGGTCAAGGGAACAGCGGCGGAA 67.74% (11154 reads)
CCAGTCCTACGTCTGGTGG-TCAAGGGAACAGCGGCGGAA 2.38% (392 reads)
CCAGTCCT-------------CAAGGGAACAGCGGCGGAA 1.95% (321 reads)
CCAGTCCTACGTCT-------CAAGGGAACAGCGGCGGAA 1.94% (319 reads)
CCAGTCCT------------TCAAGGGAACAGCGGCGGAA 1.73% (285 reads)
CCAGTCCTACGTCTG-----TCAAGGGAACAGCGGCGGAA 1.68% (277 reads)
CCAGTCCTACGTC---------AAGGGAACAGCGGCGGAA 1.48% (243 reads)
--------------------TCAAGGGAACAGCGGCGGAA 1.41% (233 reads)
CCAGTCCTACGT--------TCAAGGGAACAGCGGCGGAA 1.38% (228 reads)
CCAGTCCTACGTCT------TCAAGGGAACAGCGGCGGAA 1.06% (174 reads)
CCAGTCCTACGTCTGGT----CAAGGGAACAGCGGCGGAA 1.05% (173 reads)
CCAGTCCTACGTCTGGTGGGGTCAAGGGAACAGCGGCGGA 0.90% (148 reads)
CCAGTC----------------AAGGGAACAGCGGCGGAA 0.87% (143 reads)
CCAGTCCTACGTCTGGTG--TCAAGGGAACAGCGGCGGAA 0.86% (141 reads)
CCAGTCCTAC----------TCAAGGGAACAGCGGCGGAA 0.85% (140 reads)
CCAGTC--------------TCAAGGGAACAGCGGCGGAA 0.81% (133 reads)
CCAGTCCTACGTCTGGTGGG-CAAGGGAACAGCGGCGGAA 0.67% (110 reads)
---------------------CAAGGGAACAGCGGCGGAA 0.63% (103 reads)
CCAGT---------------TCAAGGGAACAGCGGCGGAA 0.62% (102 reads)
CCAGTCCTACGTC------GTCAAGGGAACAGCGGCGGAA 0.52% (86 reads)
CCAGTCCTA-----------TCAAGGGAACAGCGGCGGAA 0.42% (69 reads)
C---------------------AAGGGAACAGCGGCGGAA 0.42% (69 reads)
CCAGTCC------------GTCAAGGGAACAGCGGCGGAA 0.39% (65 reads)
CCAGTCCTACGTCTGGT---TCAAGGGAACAGCGGCGGAA 0.38% (63 reads)
CCAGTCCTACG--------GTCAAGGGAACAGCGGCGGAA 0.38% (62 reads)
CCAGTCCTACGT-------GTCAAGGGAACAGCGGCGGAA 0.37% (61 reads)
CCA-----------------TCAAGGGAACAGCGGCGGAA 0.36% (59 reads)
-------------------GTCAAGGGAACAGCGGCGGAA 0.35% (57 reads)
CC------------------TCAAGGGAACAGCGGCGGAA 0.32% (53 reads)
CCAGTCCT-----------GTCAAGGGAACAGCGGCGGAA 0.25% (41 reads)
bold Substitutions
Insertions
Deletions
Predicted cleavage position
